# Occlusive retinal vasculopathy with macular branch retinal artery occlusion as a leading sign of atypical hemolytic uremic syndrome – a case report

**DOI:** 10.1186/s12886-021-01820-x

**Published:** 2021-01-30

**Authors:** David Pérez González, Matias Iglicki, Shuli Svetitsky, Yaeli Bar-On, Zohar Habot-Wilner, Dinah Zur

**Affiliations:** 1 Ophthalmology Division, Tel Aviv Medical Center, Tel Aviv, Israel; 2 Private Retina Practice, University of Buenos Aires, 525 Aguirre St., 3rd floor, Apt. A, Zip code 1414 Buenos Aires, Argentina; 3 Renal and Transplant Centre, Imperial College Healthcare NHS Trust, Hammersmith Hospital, London, UK; 4 Sackler Faculty of Medicine, Tel Aviv University, Tel Aviv, Israel; 5 Bone Marrow Transplant Unit, Tel Aviv Medical Center, Tel Aviv, Israel

**Keywords:** Atypical hemolytic uremic syndrome, Occlusive retinal vasculopathy, Paracentral acute middle Maculopathy, Case report

## Abstract

**Background:**

Hemolytic Uremic Syndrome (HUS) is a rare disorder characterized by the triad of microangiopathic hemolytic anemia, thrombocytopenia, and acute renal failure, considered within the group of thrombocytic microangiopathies. Ocular complications in HUS are very rare. Here, we report an adult patient who suffered from acute onset of paracentral scotoma, caused by branch retinal artery occlusion (BRAO), as a leading symptom of atypical HUS.

**Case presentation:**

A 39-year-old healthy male was lately diagnosed with essential hypertension and mild renal impairment. He complained about acute onset of central scotoma in his left eye. Fundus examination revealed marked narrowing of retinal vessels, cotton wool spots and few retinal hemorrhages in both eyes. The patient was diagnosed with bilateral ischemic retinal vasculopathy and acute macular BRAO in his left eye. Workup revealed thrombocytopenia, worsening renal failure. Renal biopsy showed signs of chronic thrombotic microangiopathy. The patient was diagnosed with atypical HUS (aHUS) and started on plasmapheresis, together with eculizumab. As his condition continued to worsen, he was put on renal replacement therapy. Due to a persistent monoclone of IgG1, the patient underwent bone marrow biopsy which revealed Monoclonal Gammopathy of renal significance, triggering a HUS and treatment was initiated accordingly. Two months after initial presentation, the patient developed neovascularization of the optic disc (NVD) in his left eye, and was treated with 3 monthly intravitreal bevacizumab injections with complete regression of the NVD. The patient suffered from myocardial infarction in the later course and was lost for follow-up. He returned 11 months after the last bevacizumab injection because of sudden loss of vision in his left eye caused by a dense vitreous hemorrhage. Biomicroscopy revealed a new NVD in his right eye. The patient underwent panretinal photocoagulation in both eyes with regression of neovascularization. Vision improved and remained 20/20 in both eyes.

**Conclusion:**

We present a case report showing retinal ischemia can be linked with aHUS. As clinal diagnosis might be challenging, physicians should be aware of the rare ocular manifestations of this devastating multi-organ disease. In case of retinal ischemia, panretinal photocoagulation should be initiated soon to avoid blinding complications.

## Background

Hemolytic Uremic Syndrome (HUS) is a rare disorder characterized by the triad of microangiopathic hemolytic anemia, thrombocytopenia, and acute renal failure, considered within the group of thrombocytic microangiopathies [1]. The incidence of HUS is 1–2 cases per 100,000 population per year in the United States [2]. The majority of patients with typical HUS are children, and their pathophysiological entity is considered a sequel after the production of an enteropathogenic toxin, known as Shiga-like toxin (Stx) secondary to *E. coli* infection serotype 0157: H7.

Atypical HUS (aHUS) is a distinctly different illness, and is a disorder caused by hyper-activation of the alternative complement pathway due to over-activation of C3 convertases and loss of complement regulatory mechanisms [3]. aHUS is not associated with Stx. However, it can be triggered by infections, pregnancy, or drug response [1]. It can also be secondary to a monoclonal gammopathy affecting the complement system [4], opposed to typical HUS, aHUS is a very rare syndrome with an estimated incidence of 2–7 per million individuals [5, 6]. Formerly there was no specific treatment for aHUS, but it can now be treated with Eculizumab, a monoclonal antibody that binds to the terminal complement protein C5 and halts the complement cascade.

Despite having different etiologies, the clinical manifestations of HUS and aHUS are similar. Systemic involvement includes end-stage renal disease (ESRD) with cardiovascular and central nervous system complications [5]. The diagnosis based on the clinical presentation can be challenging.

Ocular complications in typical HUS are very rare and have been described in 4% of pediatric cases [7, 8]. The prevalence is lower with aHUS, with only few cases reported in the literature, including Purtscher-like retinopathy [9–11], occlusive retinopathy [12] and hypertensive choroidopathy [13].

Here, we report an adult patient who suffered from acute onset of paracentral scotoma, caused by BRAO, as a leading symptom of aHUS.

## Case presentation

A 39-year-old healthy male was evaluated for new onset of headache and dizziness. At initial presentation, blood pressure was elevated (170/100 mmHg), lab tests showed mild normocytic normochromic anemia (Hb 12.9 mg/dL), and mildly elevated creatinine (1.58 mg/dL). Causes for primary hypertension including renovascular hypertension were ruled out. Work-up for viral (HIV, VZV, HSV, HBV, HCV), and autoimmune etiologies (ANA, APLA, c-ANCA, p-ANCA, ACE, cardiolipin, beta2glycoprotein and anti-GBM) was negative. Transthoracic echocardiogram and brain MRI were normal. The patient was diagnosed with essential hypertension and mild hypertensive renal impairment and was started on amlodipine 10 mg per day.

**Fig. 1:**
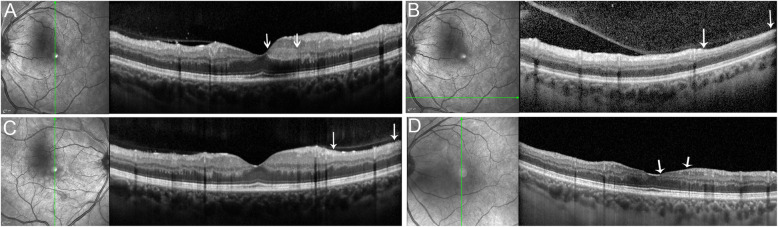
Optical Coherence Tomography at Presentation and Follow-up. **a**-**b** Left eye at presentation: Vertical scan showing paracentral acute medial maculopathy, appearing as a hyperreflective band involving the the inner nuclear layer superior to the fovea (**a**, arrows) and a horizontal inferior scan showing inner retinal thinning, indicating previous retinal ischemia (**b**, arrows). **c** Vertical scan of the right eye at presentation showing mild thinning of the retinal nerve fiber and ganglion cell layer, compatible with previous inner ischemia. **d** Left eye at last visit showing thinning of the affected INL in the area of the BRAO (arrows)

**Fig. 2:**
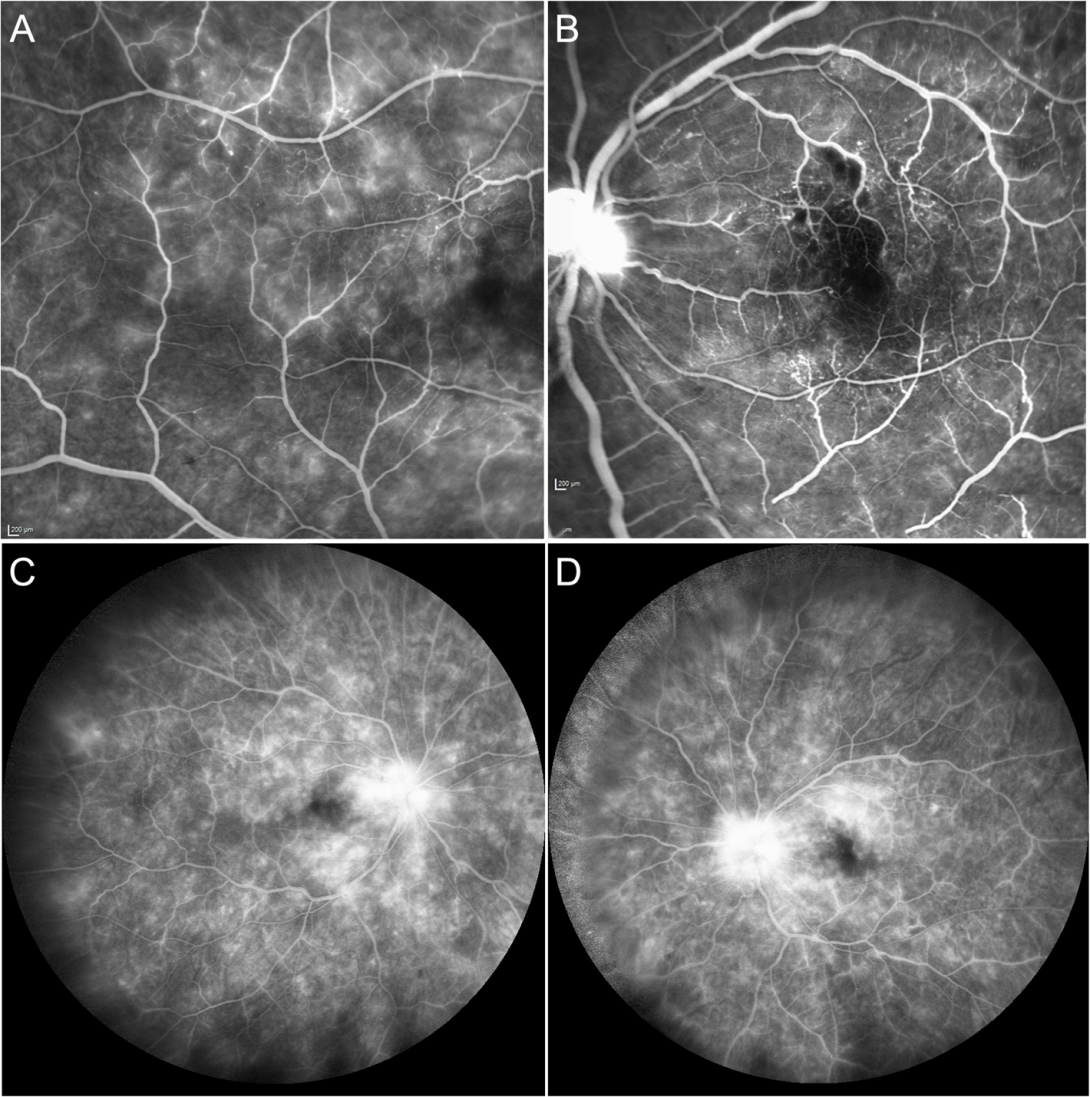
Fluorescein Angiography – Bilateral Occlusive Vasculopathy at Presentation. **a** Fluorescein angiography (FA) of the right eye temporal to the macular showing leakage from retinal capillaries, and teleangiectatic vessels (3.30 min). **b** FA of the left eye showing hypofluorescence superior and involving the fovea, compatible with hypoperfusion due to branch retinal artery occlusion. Note leakage from retinal capillaries and optic disc (1.18 min). **c**-**d** Late phase showing capillary leakage, peripheral capillary nonperfusion and marked hot discs (8 min)

Two months after initial presentation, the patient complained about acute onset of central scotoma in his left eye. The ophthalmologic examination revealed, best corrected visual acuity (BCVA) 20/20 in each eye. There was no relative afferent pupillary defect. Visual field testing showed an inferotemporal paracentral scotoma in his left eye. Intraocular pressure was 15 and 17 mmHg, in his right and left eye, respectively. Anterior chambers and vitreous were clear in both eyes. Fundus examination revealed marked narrowing of retinal vessels, cotton wool spots and few retinal hemorrhages in both eyes. The left eye showed retinal whitening superior to the fovea, compatible with acute macular BRAO. Optical coherence tomography (OCT) showed signs of retinal ischemia superior to the fovea in his left eye and we saw areas of extramacular retinal thinning compatible with previous branch artery occlusions in both eyes, which have not been noticed by the patient (Fig. 1). Fluorescein angiography (FA) showed macular and peripheral capillary non-perfusion, leakage from retinal vessels, and from the optic nerves on late frames in both eyes (Fig. 2). The patient was diagnosed with bilateral ischemic retinal vasculopathy and macular BRAO in his left eye. Although the ocular presentation included signs of hypertensive retinopathy, the severity of the retinal disease did not match the systemic condition of essential hypertension in a young patient. With a differential diagnosis of vasculitis, he was started on prednisone 1 mg per kg, and laboratory tests were repeated.

Further workup revealed now thrombocytopenia (88.000), worsening renal failure (creatinine 3.71 mg/dL), elevated LDH (740), a low level of haptoglobin, a few red cell fragments on blood film, all susggesting a Thrombotic microangiopathic process. A stool culture and PCR for Shiga toxin-producing *Escherichia coli* was negative and thrombotic thrombocytopenic purpura was ruled out by normal ADAMTS13 activity and lack of an ADAMTS13 antibody.

Further investigation of the progressive renal failure, revealed a small monoclone of IgG lambda serum protein electrophoresis, with a normal Free Light chain ratio, and a negative Bence jones in the urine (Bence Jones protein is a monoclonal globulin protein or immunoglobulin light chain found in the urine, with a molecular weight of 22–24 kDa [14]. Detection of Bence Jones protein may be suggestive of multiple myeloma). On Bone marrow aspiration there was a normocellular marrow with 2% monoclonal plasma cells, incompatible with active multiple myeloma.

The patient underwent renal biopsy showing signs of chronic thrombotic microangiopathy, and the patient was diagnosed with aHUS. Genetic testing for aHUS, showed no clinically relevant mutations, and HLA B-5 testing for Behcet’s disease was negative, as was the repeat panel of rheumatic diseases. The patient was started on Plasmapheresis, together with eculizumab. Under this combination there was an initial improvement, but after more than 2 months of this treatment the patient’s condition continued to deteriorate and he was started on renal replacement therapy.

At that stage, repeat tests were taken, showing the same monoclone and similar numer of plasma cells in the bone marrow. After several multidisciplinary discussions he was diagnosed with Monoclonal Gammopathy of renal significance, triggering aHUS. The patient was started on a Bortezomib based induction (Bortezomib, cyclophosphamide and dexamethasone), hoping this would slow the process.

Repeated FA 1 month after initial presentation showed marked improvement of vessel leakage, with slow improvement of his renal function, and stabilization of the haemolytic process. After 3 cycles, due to slow renal and hematological response, it was decided to move to second line treatment with Daratumumab (anti CD38 monoclonal antibody), lenalidomide and dexamethasone (DRd).

**Fig. 3:**
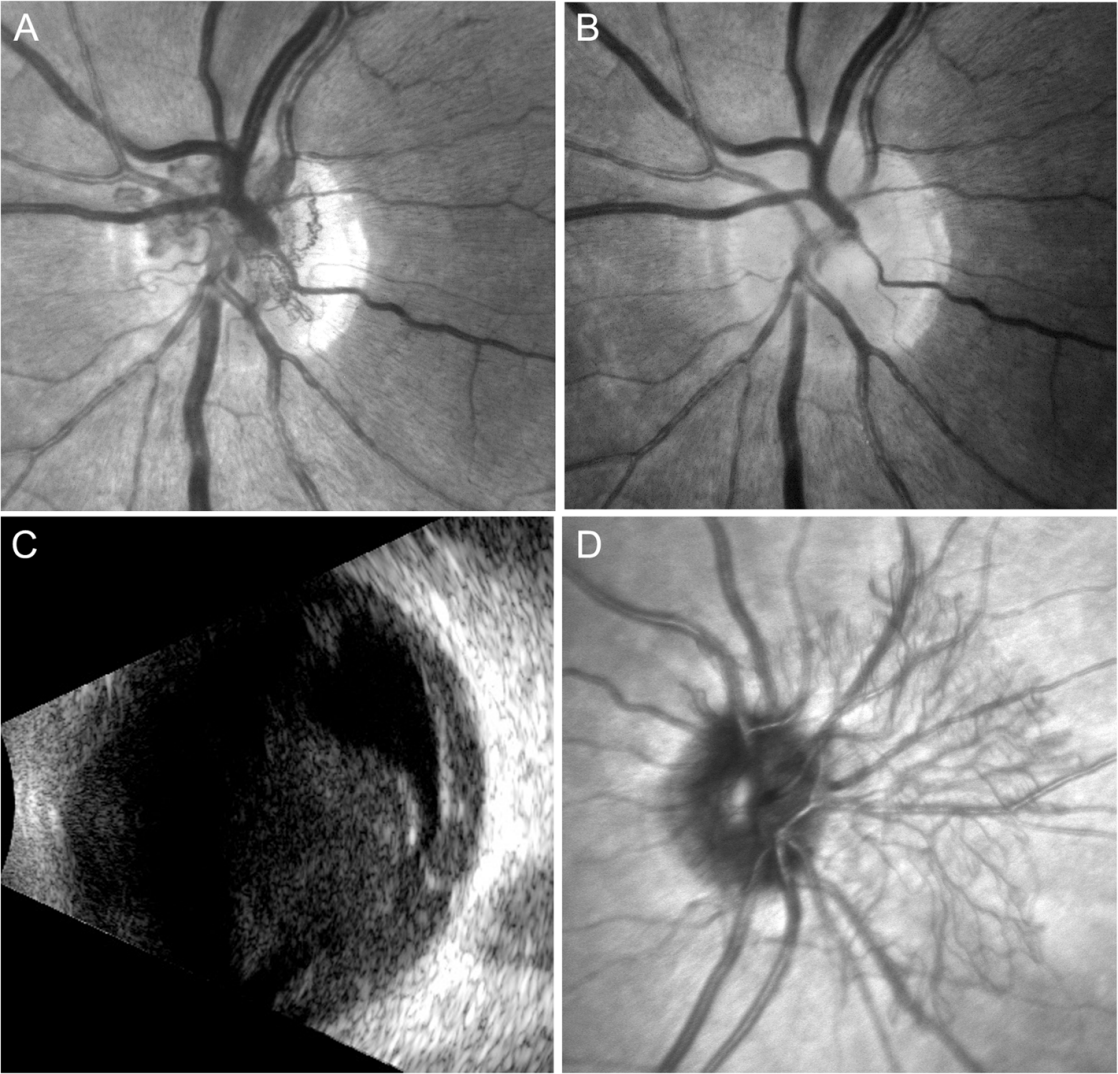
Neovascular Complications. **a** Left eye with neovascularization of disc (NVD). **b** After 3 intravitreal injections of bevacizumab, the NVD regressed completely. **c** Ultrasound B-scan showing vitreous opacity originating from the optic disc, caused by recurrence of the NVD. **d** New onset of NVD in the right eye

**Fig. 4:**
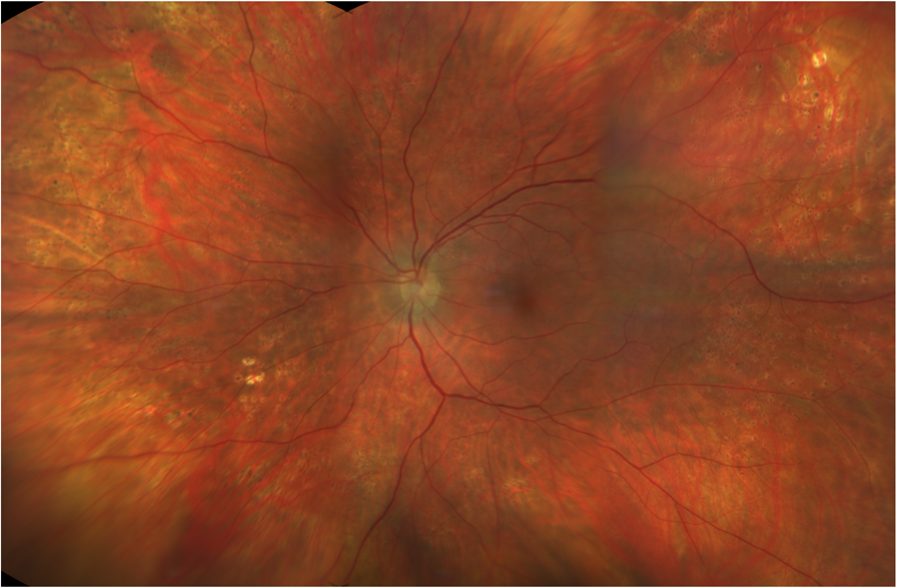
Color Fundus Photography at Last Visit. Wide-field fundus photography of the left eye at last visit showing regression of the NVD, mildly narrowed retinal arteries and panretinal photocoagulation scars in the periphery

**Fig. 5:**
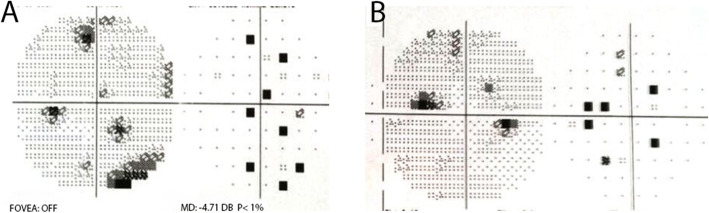
Visual Field Exam. 30–2 visual field exam revealed peripheral defects in the right eye (**a**) and inferotemporal paracentral scotoma and peripheral defects in the left eye (**b**)

At this stage, neovascularization of the optic disc (NVD) in his left eye was diagnosed (Fig. 3a). The patient did not give consent for panretinal photocoagulation (PRP) because of fear for peripheral visual field impairment. However, he agreed and received 3 monthly intravitreal bevacizumab injections (1.25 mg/ml). One month after the last injection we noticed complete regression of the NVD (Fig. 3b). During the later course, the patient suffered from myocardial infarction, a well known complication of aHUS [7], and underwent percutaneous transluminal coronary angioplasty with stent implantation. Due to his systemic condition he was lost for ophthalmological follow-up and returned 11 months after the last bevacizumab injection because of sudden loss of vision in his left eye. BCVA was 20/20 in his right eye and 20/400 in his left eye. Biomicroscopy revealed a new NVD in his right eye and a dense vitreous hemorrhage in his left eye (Fig. 3). Ocular ultrasound showed vitreous opacities compatible with hemorrhage, evolving from a NVD (Fig. 3). The patient underwent PRP in his right eye with complete regression of the neovascularization. After one more bevacizumab injection at this time and gradual resolution of the vitreous hemorrhage, PRP was performed in his left eye. The NVD regressed and vision returned to 20/20 (Fig. 4). Visual field revealed inferotemporal paracentral scotoma in the left eye and peripheral defects in both eyes (Fig. 5).

Under the above mentioned treatment of DRd, the patient’s monoclone disappeared, and the thrombotic microangiopathy process was halted with weaning of renal replacement therapy, and almost complete normalization of the platelets, with normal LDH and haptoglobin, and no need for renal replacement therapy.

## Discussion and conclusion

Atypical HUS is a very rare, complex and potentially fatal condition with an initial mortality rate of up to 25%, dependent on the genetic mutation [15]. The typical presentation is with the triad of hemolytic anemia, thrombocytopenia, and renal dysfunction. In our case, the patient did not present the typical manifestation of aHUS initially. After extensive work up, laboratory findings were initially interpreted as complications of essential hypertension. The sudden onset of scotoma in the patient’s left eye led to the diagnosis of acute retinal ischemia with occlusive vasculopathy, and after repeated workup to the final diagnosis of aHUS.

Extrarenal manifestations are reported in 20% of aHUS cases [7]. As the initial diagnosis can be challenging, as in our case, it is important to be aware of these severe extrarenal organ involvement, in order to ensure timely diagnosis and prompt initiation of treatment.

Ocular complications of aHUS are exceedingly rare. The different retinal manifestations described in the literature all result from occlusive (i.e. thrombotic) microangiopathy [10–12, 16]. In our case, FA showed macular and peripheral ischemia with mild leakage from small retinal vessels. Due to the extensive peripheral ischemia the patient developed proliferation of neovascularization of both optic discs. In the left eye, NVD was noted only 4 months after the diagnosis of aHUS was established. Similarly, Lin et al. described a case of aHUS with severe capillary nonperfusion who developed bilateral proliferative retinopathy and ischemic optic neuropathy as soon as 2 months after diagnosis of aHUS [12]. As in our case, the patient was initially treated with an intravitreal anti-VEGF injection. However, his condition worsened, and he developed bilateral tractional retinal detachment and underwent vitrectomy. The extent of capillary nonperfusion and the potential for early neovascular complications highlight the need for early intervention with panretinal photocoagulation in order to prevent blinding complications.

aHUS is characterized by an uncontrolled systemic complement activation. While the mechanism has not been fully elucidated, there is agreement that complement components mediate activation of leukoembolization and exert the coagulation cascade in endothelial cells, augmenting the prothrombotic state in retinal vessels [5].

Eculizumab is a monoclonal antibody directed against complement protein C5. The introduction of eculizumab has revolutionized the care of aHUS patients and dramatically reduced the rate of disease recurrence after kidney transplants [17]. Eculizumab has been shown beneficial in the resolution of retinal complications, as well [11, 16]. In our case, kidney function did not improve under eculizumab therapy and hence the therapy was discontinued. As in our case, there have been cases of aHUS in the presence of monoclonal gammopathy, in which the paraprotein activates the complement system [18].

Apart from ocular involvement, our patient developed central nervous system (CNS), cardiac, and hematologic complications of aHUS. CNS involvement has been reported in 10% of patients and cardiac complications can occur in 3–10% of patients [19].

In conclusion, we presented a case report showing retinal ischemia can be linked with aHUS. As clinal diagnosis might be challenging, physicians should be aware of the rare ocular manifestations of this devastating multi-organ disease. In case of retinal ischemia, panretinal photocoagulation should be initiated soon to avoid blinding complications.

## Data Availability

All data generated or analysed during this study are included in this published article.

## Abbreviations

aHUS: atypical hemolytic uremic syndrome
BCVA: Best corrected visual acuity
FA: Fluorescein angiography
NVD: Neovascularization of disc
OCT: Optical coherence tomography
